# Sugar for the trip: Pollen tube development requires glycosylated flavonols

**DOI:** 10.1093/plphys/kiaf580

**Published:** 2025-11-09

**Authors:** María Flores-Tornero

**Affiliations:** Assistant Features Editor, Plant Physiology, American Society of Plant Biologists; Departament de Biologia Vegetal, Facultat de Ciències Biològiques, Universitat de València, Valencia, Burjassot 46100, Spain

In addition to producing primary metabolites to maintain our planet's food chains, plants also produce secondary or specialized metabolites that perform different functions and provide them with specific advantages.

Flavonols are a special type of secondary metabolites with diverse functions and structures. These molecules can be modified in several ways to produce different structures tightly related to their role. Glycosylation is the most common chemical modification, and it is catalyzed by different glycosyltransferases that are expressed in many different parts of the plant. Composition and content of flavonol glycosides vary across plant species and organs. For example, they are needed for pollen tube germination, as their absence leads to abnormal pollen in tobacco (Ylstra et al. 1992) or rice (Wang et al. 2020). Moreover, it is known that high flavonol content in pollen enhances thermotolerance during pollen tube growth (Postiglione et al. 2024).

In this issue of *Plant Physiology*, Shin and colleagues explored the role of a tomato flavonol glycosidase specifically expressed in pollen and its physiological role in pollen tube germination. By using high-performance liquid chromatography, the authors analyzed the flavonol profile of several organs of tomato, discovering that pollen grains show a unique flavonol pattern with a predominant compound. Tandem mass spectrometry analysis and comparisons with a previous study on petunia pollen grains (Vogt and Taylor 1995) guided Shin and colleagues to discover that this compound was kaempferol 3-O-glucosyl(1 → 2)galactoside (K-gal-glu), hereafter K2.

Interestingly, petunia pollen also showed another abundant flavonol glycoside, quercetin 3-O-glucosyl(1 → 2) galactoside (Q2), that was absent not only in tomato pollen but also in other more recently diverged Solanacea species like chili pepper or potato. This finding indicates a pollen-specific flavonol profile, maintaining Q2 in some species and losing it in others, like tomato.

They knew that production of the glycosylated flavonol K2 involves the sequential action of a galactosyltransferase and a glucosyltransferase, but in tomato there are many predicted enzymes of these classes (Tomato Genome Consortium 2012) without any functional confirmation. Therefore, the authors performed a phylogenetic analysis with the amino acid sequence of tomato-predicted glycosyltransferases with those already characterized in other species like Arabidopsis (Jones et al. 2003), petunia (Miller et al. 1999), or maize (Taylor and Briggs 1990) to create clusters and identify putative candidates for these enzymes in tomato. As a result, Shin and colleagues identified 2 potential genes, one absent in pollen (Solyc10g083440, hereafter 78-A) and the other highly expressed there (Solyc07g006720, hereafter 78-B) (Fig. A).

**Figure.**
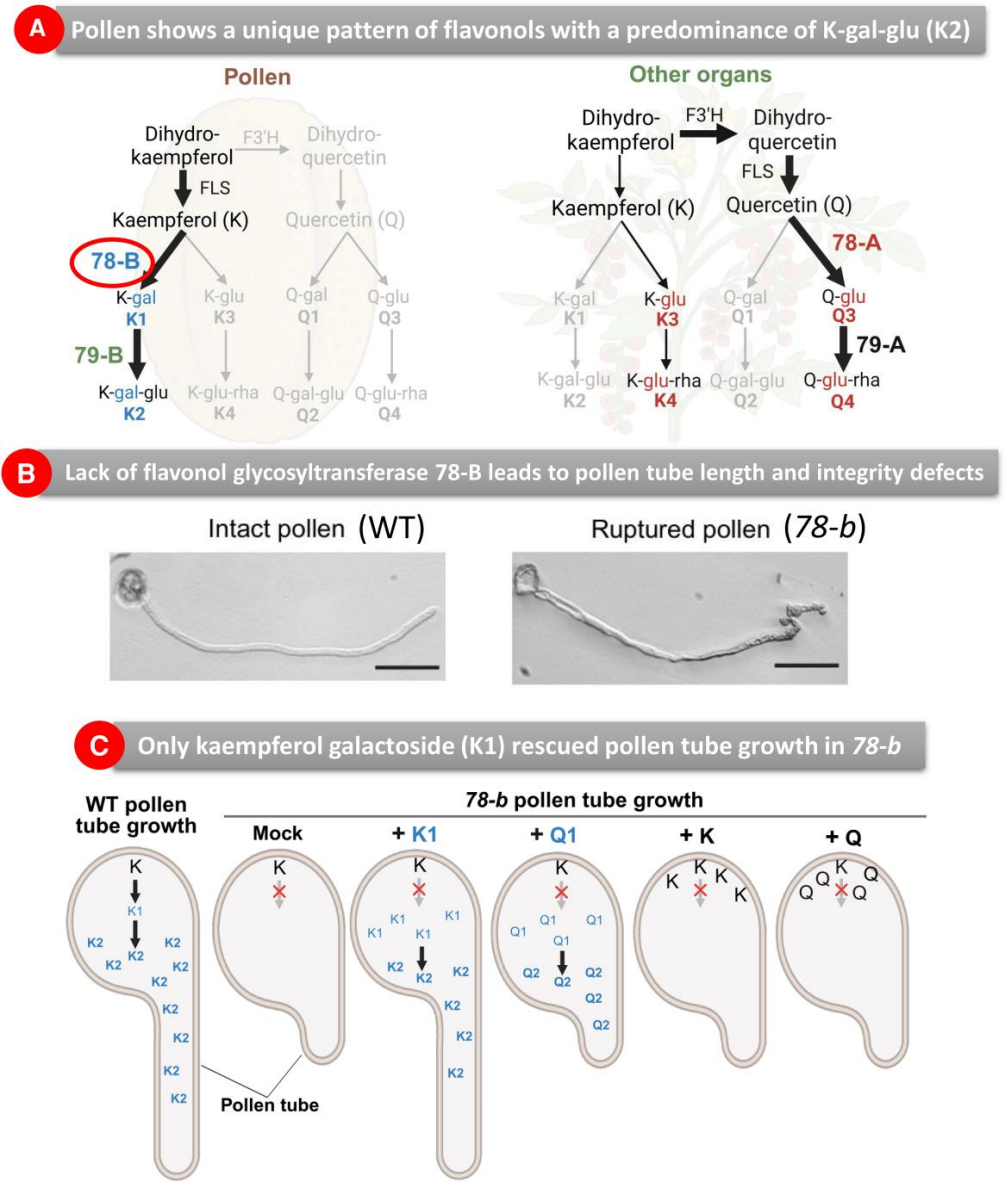
Key findings related to flavonol glycoside kaempfero l3-O-glucosyl(1→2)galactoside (K2). **A)** K2 and the enzymes that produce it, glycosyltransferases 78-B and 79-B, are specifically expressed in pollen grains. **B)** Absence of 78-B significantly alters pollen tube growth and integrity. **C)** A chemical complementation assay showed that only kaempferol galactoside (K1) can revert this abnormal pollen tube phenotype. Figures were taken and adapted from Shin et al. (2025).

When testing their enzymatic activities, the authors discovered that 78-B shows only galactosyltransferase, whereas 78-A contains the 2 necessary glycosyltransferases to produce K2. The absence of 78-A in pollen and the lack of glucosyltransferase in 78-B suggested the presence of an additional enzyme in pollen to catalyze the final step to produce K2. This missing glucosyltransferase was identified in Solyc10g008860, hereafter 79-B, clustering with a characterized glucosyltransferase from petunia.

This lack of glucosyltransferase activity in 78-B compared with 78-A prompted the authors to compare their amino acid sequences with those of characterized glycosyltransferase homologues from other plant species (Cui et al. 2016), resulting in the identification of 4 key residues putatively responsible of this restricted activity. To check the relevance of these amino acids, directed mutations on those residues in 78-B coupled to in vitro enzymatic activity essays led to the artificial acquisition by 78-B of the missing glycosyltransferase activity that was needed to produce K2.

To get functional insights on the role of 78-A and 78-B in plant development, Shin and colleagues generated *78-a* and *78-b* mutants by CRISPR/Cas9 and confirmed the absence of K2 in their pollen. No effect on development or vegetative growth was observed, but pollen tube length and its integrity were significantly altered (Fig. B).

To close the circle, the authors tested different pollen-specific flavonols in the pollen germination medium to examine whether they could revert the alterations observed in *78-b* germinating pollen tubes (Fig. C). They observed that only when kaempferol galactosides (K1) were added to the pollen germination media, abnormal pollen tube growth was restored.

Taken together, this work highlights the specific role of individual flavonol glycosidases on a sensitive process like pollen tube growth and development, a crucial step to ensure fertilization. However, it is known that female tissue also plays a role in pollen tube growth and guidance by pollen tube–pistil interactions (Song et al. 2024). Therefore, an interesting experiment would be to germinate pollen from *78-a* and *78-b* through pistil sections (semi-in vivo germinations) to observe if female secretions are playing a compensatory role on helping pollen tube germination in these mutants.

## Data Availability

No new data were generated or analyzed in support of this research.
